# A robust approach for multi-type classification of brain tumor using deep feature fusion

**DOI:** 10.3389/fnins.2024.1288274

**Published:** 2024-02-19

**Authors:** Wenna Chen, Xinghua Tan, Jincan Zhang, Ganqin Du, Qizhi Fu, Hongwei Jiang

**Affiliations:** ^1^The First Affiliated Hospital, and College of Clinical Medicine of Henan University of Science and Technology, Luoyang, China; ^2^College of Information Engineering, Henan University of Science and Technology, Luoyang, China

**Keywords:** brain tumor classification, deep learning, transfer learning, ResNet101, DenseNet121, EfficientNetB0, feature fusion

## Abstract

Brain tumors can be classified into many different types based on their shape, texture, and location. Accurate diagnosis of brain tumor types can help doctors to develop appropriate treatment plans to save patients’ lives. Therefore, it is very crucial to improve the accuracy of this classification system for brain tumors to assist doctors in their treatment. We propose a deep feature fusion method based on convolutional neural networks to enhance the accuracy and robustness of brain tumor classification while mitigating the risk of over-fitting. Firstly, the extracted features of three pre-trained models including ResNet101, DenseNet121, and EfficientNetB0 are adjusted to ensure that the shape of extracted features for the three models is the same. Secondly, the three models are fine-tuned to extract features from brain tumor images. Thirdly, pairwise summation of the extracted features is carried out to achieve feature fusion. Finally, classification of brain tumors based on fused features is performed. The public datasets including Figshare (Dataset 1) and Kaggle (Dataset 2) are used to verify the reliability of the proposed method. Experimental results demonstrate that the fusion method of ResNet101 and DenseNet121 features achieves the best performance, which achieves classification accuracy of 99.18 and 97.24% in Figshare dataset and Kaggle dataset, respectively.

## 1 Introduction

In recent years, the rising incidence and mortality rates of brain tumor diseases have posed significant threats to human well-being and life (Satyanarayana, 2023). Because of the different causes and locations of brain tumors, the treatment methods for brain tumors are very different. Additionally, the severity of lesions significantly impacts the efficacy of treatment methods. Therefore, it is very important to determine the type and severity of brain tumor lesions prior to treatment development. With the development of modern technology, Computer-Aided Diagnosis (CAD) technology plays an increasingly important role in the medical diagnosis process (Fujita, 2020; Gudigar et al., 2020; Sekhar et al., 2022). The diagnosis and analysis of brain tumor magnetic resonance imaging (MRI) images by physicians based solely on personal experience is not only inefficient but also subjective and prone to errors, leading to misleading results (Chan et al., 2020; Arora et al., 2023). Consequently, enhancing the efficiency and accuracy of computer-aided diagnosis for brain tumors has emerged as a prominent research hotspot in the field of brain tumor-assisted diagnosis.

Traditionally, the classification method of medical images consists of several stages, including image pre-processing, image segmentation, feature extraction, feature selection, training of classifiers and image classification (Muhammad et al., 2021; Yu et al., 2022). Nevertheless, in recent years, with the emergence of deep learning theory, more and more researchers applied the deep learning theory into medical image processing (Maurya et al., 2023). Deep learning has been employed widely in the analysis and diagnosis of diverse diseases (Cao et al., 2021; Gu et al., 2021; Lin et al., 2022; Yang, 2022; Yao et al., 2022; Zolfaghari et al., 2023). Convolutional Neural Networks (CNNs) are widely recognized as one of the most prominent deep learning techniques. By utilizing the images as input, CNNs mitigate the issue of low classification accuracy resulting from the selection of unrepresentative features by humans.

Medical images are usually difficult to obtain, and the amount of image data is relatively small (Shah et al., 2022). Although training an effective deep learning model typically necessitates a substantial amount of data, transfer learning can address the issue of limited dataset size and expedite the training process. Therefore, transfer learning has been widely used in the medical field (Yu et al., 2022). Yang et al. (2018) utilized AlexNet and GoogLeNet for glioma grade classification. Experimental results demonstrated that CNNs trained using transfer learning and fine-tuning were employed for glioma grading, achieving improved performance compared to traditional machine learning methods reliant on manual features, as well as compared to CNNs trained from scratch. Swati et al. (2019) and Zulfiqar et al. (2023) employed VGG19 and EfficientNetB2, respectively for the classification of brain tumors. Arora et al. (2023) examined the classification performance of 14 pre-trained models for the identification of skin diseases. DenseNet201 obtained superior classification performance, achieving an accuracy of 82.5%. Meanwhile, ResNet50 exhibits the second-highest classification accuracy at 81.6%. Aljuaid et al. (2022), ResNet 18, ShuffleNet, and Inception-V3Net models were used to classify breast cancer, with ResNet 18 showing excellent performance with an accuracy of 97.81%.

However, only relying on a single model often results in over-fitting on the training set and poor generalization on the test set, in turn to diminish the model’s robustness. Therefore, in this paper, to addresses the limitations associated with only relying on a single model, model integration techniques are proposed. In this paper, three pre-trained models namely ResNet101, DenseNet121, and EfficientNetB0 are used to extract the features of brain tumor images. Subsequently, the extracted features are fused using a summation method, followed by classification of the fused features. The main contributions of this paper are as follows:

1. An image classification method for brain tumors based on feature fusion is proposed.
2. The feature outputs of the three pre-trained models were adjusted to have consistent dimensions.
3. Feature fusion was accomplished through summation.
4. The validity of the method was verified on two publicly available datasets including Figshare dataset (Cheng et al., 2015) referred to as dataset 1, and Kaggle dataset (Bhuvaji et al., 2020) referred to as dataset 2, and the model outperformed other state-of-the-art models.

## 2 Related work

There have been many studies on the classification of brain tumors.

Alanazi et al. (2022) constructed a 22-layer CNN architecture. Initially, the model underwent training with a large dataset utilizing binary classification. Subsequently, the model’s weights were adjusted, and it was evaluated on dataset 1 and dataset 2 using migration learning. The model achieved accuracy of 96.89 and 95.75% on dataset 1 and dataset 2, respectively. Hammad et al. (2023) constructed a CNN model with 8 layers. The model achieved an accuracy of 99.48% for binary classification of brain tumors and 96.86% for three-class classification. Liu et al. (2023) introduced the self-attention similarity-guided graph convolutional network (SASG-GCN) model to classify multi-type low-grade gliomas. The model incorporates a convolutional depth setting signal network and a self-attention-based method for chart construction on a 3D MRI water surface, which achieved an accuracy of 93.62% on the TCGA-LGG dataset. Kumar et al. (2021) employed the pre-trained ResNet50 model for brain tumor classification, achieving a final accuracy of 97.48% on dataset 1. Swati et al. (2019) presented an exposition on the merits and demerits of conventional machine learning and deep learning techniques. They introduced a segmented fine-tuning approach leveraging a pre-trained deep convolutional neural network model. Through fine-tuning, they achieved an accuracy of 94.82% on dataset 1 using the VGG19 architecture. Ghassemi et al. (2020) employed a pre-trained generative adversarial network (GAN) for feature extraction in the classification of brain tumors. The experiment was conducted on dataset 1, yielding an accuracy of 95.6%. Saurav et al. (2023) introduced a novel lightweight attention-guided convolutional neural network (AG-CNN). This network incorporates a channel attention mechanism. The model achieves accuracies of 97.23 and 95.71% on dataset 1 and dataset 2, respectively.

Integration through models is a feasible solution. In Hossain et al. (2023), an ensemble model IVX16 was proposed based on the average of the classification results of three pre-trained models (VGG16, InceptionV3, Xception).The model achieved a classification accuracy of 96.94% on dataset 2. A comparison between IVX16 and Vison Transformer (ViT) models reveals that IVX16 outperforms the ViT models. Tandel et al. (2021) presented a method of majority voting. Firstly, five pre-trained convolutional neural networks and five machine learning models are used to classify brain tumor MRI images into different grades and types. Next, a majority voting-based ensemble algorithm is utilized to combine the predictions of the ten models and optimize the overall classification performance. In Kang et al. (2021), nine pre-trained models including ResNet, DenseNet, VGG, AlexNet, InceptionV3, ResNeXt, ShuffleNetV2, MobileNetV2, and MnasNet were employed. The pre-trained models were utilized to extract features, which were then forwarded to a machine learning classifier. From the extracted features, three deep features with excellent performance were selected and concatenated along the channel dimension. The resulting feature representation was subsequently sent to both the machine learning classifier and fully connected (FC) layer. On dataset 2, the model achieved an accuracy of 91.58%. Alturki et al. (2023) employed a voting-based approach to classify brain tumors as either healthy or tumorous. They utilized a CNN to extract tumor features, and employed logistic regression and stochastic gradient descent as the classifiers. To achieve high accuracy of tumor classification, a soft voting method was employed.

Furthermore, the combination of CNNs and machine learning classifiers offers the potential ways to enhance the model’s performance. Sekhar et al. (2022), image features were extracted using GoogLeNet, and feature classification was performed using both support vector machines (SVM) and K-Nearest Neighbor (KNN). Ultimately, KNN outperformed SVM, achieving a model accuracy of 98.3% on dataset 1. Deepak and Ameer (2021) employed a hybrid approach combining CNN and SVM to effectively classify three distinct types of brain tumors. The researchers introduced a CNN architecture comprising five convolutional layers and two fully-connected layers. Subsequently, they extracted features from the initial fully connected layer of the designed CNN model, and ultimately performed classification using SVM. Remarkably, this approach achieved an impressive classification accuracy of 95.82% on dataset 1. Özyurt et al. (2019), the researchers utilized a hybrid approach called Neutrosophy and Convolutional Neural Network (NS-CNN) to classify tumor regions that were segmented from brain images into benign and malignant categories. Initially, the MRI images undergo segmentation employing the Neutral Set Expert Maximum Fuzzy Determination Entropy (NS-EMFSE) method. Subsequently, the features of the segmented brain images are extracted through a CNN and then classified using SVM and K-Nearest Neighbors (KNN) classifiers. The experimental results demonstrated that the utilization of CNN features in conjunction with SVM yielded superior classification performance, achieving an average accuracy of 95.62%. Gumaei et al. (2019) introduced the classification method of brain tumors based on the hybrid feature extraction method of regularized extreme learning machine (RELM). In this paper, the mixed feature extraction method is used to extract the features of brain tumors, and RELM is used to classify the types of brain tumors. This method achieves 94.233% classification accuracy on dataset 1. Öksüz et al. (2022) introduced a method that combines deep and shallow features. Deep features of brain tumors were extracted using pre-trained models: AlexNet, ResNet-18, GoogLeNet, and ShuffleNet. Subsequently, a shallow network is developed to extract shallow features from brain tumors, followed by fusion with the deep features. The fused features are utilized to train SVM and KNN classifiers. This method achieves a classification accuracy of 97.25% on dataset 1. In their work, Demir and Akbulut (2022) developed a Residual Convolutional Neural Network (R-CNN) to extract profound features. Subsequently, they applied the L1-Norm SVM ReliefF (L1NSR) algorithm to identify the 100 most discriminative features and utilized SVM for classification. The achieved classification accuracies for 2-categorized and 4-categorized data were 98.8 and 96.6%, respectively.

Moreover, the hyperparameters of the model can be optimized through the utilization of an optimization algorithm. Ren et al. (2023), the study employed preprocessing, feature selection, and artificial neural networks for the classification of brain tumors. Furthermore, the authors utilized a specific optimization algorithm known as water strider courtship learning to optimize both the feature selection and neural network parameters. The effectiveness of the proposed method was evaluated on the “Brain-Tumor-Progression” database, obtaining a final classification accuracy of 98.99%. SbDL was utilized by Sharif et al. (2020) for saliency map construction, while deep feature extraction was performed using the pre-trained Inception V3 CNN model. The connection vector was optimized using Particle Swarm Optimization (PSO) and employed for classification with the softmax classifier. The proposed method was validated on Brats2017 and Brats2018 datasets with an average accuracy of more than 92%. In Nirmalapriya et al. (2023), employed a combination of U-Net and CFPNet-M for segmenting brain tumors into four distinct classes. The segmentation process was conducted using the Aquila Spider Monkey Optimization (ASMO) to optimize segmentation model and the Spider Monkey Optimization (SMO), Aquila Optimizer (AO), and Fractional Calculus (FC) optimized SqueezeNet models. The model achieved a tested accuracy of 92.2%. The authors introduced a model, referred to in Nanda et al. (2023) as the Saliency-K-mean-SSO-RBNN model. This model comprises the K-means segmentation technique, radial basis neural network, and social spider optimization algorithm. The tumor region is segmented using the k-means clustering method. The segmented image then undergoes feature extraction through multiresolution wavelet transform, principal component analysis, kurtosis, skewness, inverse difference moment (IDM), and cosine transforms. The clustering centers are subsequently refined using the social spider optimization (SSO) algorithm, followed by processing the feature vectors for efficient classification using the radial basis neural network (RBNN). The final model achieves classification accuracies of 96, 92, and 94% on the three respective datasets.

## 3 Materials and methods

This paper utilizes three pre-trained models, namely ResNet101, DenseNet121, and EfficientNetB0. The outputs of these models are adjusted to ensure consistent data size, and then the extracted features from these models are fused. Subsequently, feature classification is performed. To achieve consistent output from the feature extraction modules across all models, we harmonized the feature extraction modules of EfficientNetB0 and ResNet101 with DenseNet121 by utilizing a 1 × 1 convolutional layer.

### 3.1 Datasets and Preprocessing

The study employed two datasets. Dataset 1, introduced by Cheng et al. (2015), is a publicly available dataset comprising 3,064 T1 MRI images. It includes three different types of brain tumors: glioma (1,426 images), meningioma (708 images), and pituitary tumor (930 images). Dataset 2, a widely used open-source dataset (Bhuvaji et al., 2020), encompasses 3,264 MRI images which consist of four categories: glioma (926 images), meningioma (937 images), pituitary tumor (901 images), and normal (500 images).

The MRI data consists of two-dimensional images with a size of 512 × 512. However, the input of the pre-training model is necessary to be RGB image. Therefore, the images were resized to dimensions of 224 × 224 × 3. Furthermore, the min-max normalization method was adopted to scale the intensity values of the image to the range of [0, 1]. The dataset 2 was processed in the same way. We divided the dataset into a training set and a test set with a ratio of 8:2.

### 3.2 Architecture of the proposed method

Transfer learning is a kind of machine learning technique, which leverages the knowledge acquired during training on one problem to train on another task or domain. The transfer learning approach, which utilizes pre-trained network knowledge obtained from extensive visual data, is very advantageous in terms of time-saving and achieving superior accuracy compared with training a model from scratch (Yu et al., 2022; Arora et al., 2023).

ResNet, DenseNet and EfficietNet have been proved to be very effective brain tumor classification models (Zhang et al., 2023; Zulfiqar et al., 2023). The accuracy of brain tumor classification of VGG19 and ResNet50 is 87.09 and 91.18%, respectively (Zhang et al., 2023). The accuracy of GoogLeNet is 94.9% (Sekhar et al., 2022). We also have tested the ability of ResNet101 and EfficientNetB0 for brain tumor classification, whose accuracy is 96.57, 96.41%, respectively. The comparison shows that ResNet101, DenseNet121 and EfficientNetB0 are more accurate, so they are chosen as the basic models.

**Figure 1:**
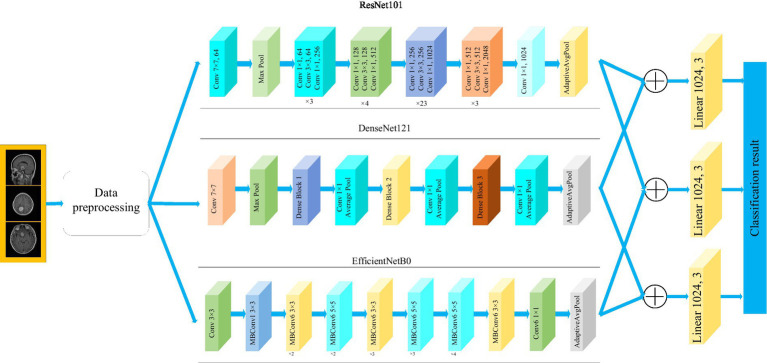
Framework diagram of the proposed methodology.

Figure 1 depicts the framework of the proposed method in this paper. Firstly, the brain tumor data was processed and the images were adjusted. Secondly, features are extracted from brain tumor images using pre-trained models. Finally, the extracted features are then aggregated for feature fusion, followed by classification. Specifically, ResNet101, DenseNet121, and EfficientNetB0 serve as pre-trained models. The outputs of the ResNet101 and EfficientNetB0 feature extraction layers are adjusted to dimensions of (1,024, 7, 7). Brain tumor feature fusion is accomplished by pairwise summation of the extracted features. Finally, the fused features are classified using a linear classifier.

### 3.3 Pre-trained models

As a fundamental component of neural network architecture, the convolutional layer extracted features by sliding a fixed-size convolutional kernel over the original image and performing multiplication operations between the kernel parameters and the image. To achieve different effects, the convolution operation relies on additional parameters, primarily the step size, padding, and size of the convolution kernel. The size of the output features from the convolutional layer can be calculated using Equation (1).


(1) $$\begin{array}{l} H_{\mathrm{out}}=\frac{H_{\mathrm{in}}+2\times \mathrm{padding}\left[0\right]-\ker nel:\mathrm{size}\left[0\right]}{\mathrm{stride}\left[0\right]}+1\\ W_{\mathrm{out}}=\frac{W_{\mathrm{in}}+2\times \mathrm{padding}\left[1\right]-\ker nel:\mathrm{size}\left[1\right]}{\mathrm{stride}\left[1\right]}+1 \end{array}$$

where *H*_in_ and *W*_in_ represent the dimensions of the input data, padding refers to the number of zero-padding layers, *Kernel_size* represents the dimensions of the convolution kernel. And stride represents the step size of the convolution operation. The formula indicates that when the *kernel_size* is set to (1,1), the stride is set to 1 and padding is set to 0, the output dimension of the convolutional layer remains unchanged.

#### 3.3.1 ResNet101

**Figure 2:**
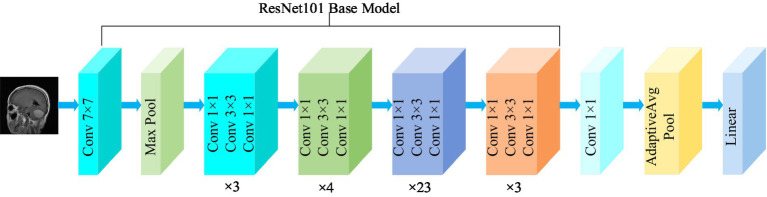
Structure of the ResNet101 model.

Residual network (ResNet) is a widely recognized and straightforward model used for deep learning tasks, particularly in image recognition (He et al., 2016). Previously, as the number of network layers increases, a common issue of vanishing gradients may arise, resulting in performance saturation and degradation of the model. Deep residual networks address this issue by incorporating jump connections between layers to mitigate information loss. The core idea of the deep residuals network is to add a path parallel to the main convolution path, which combines the features from the subsequent convolution layer with those from the previous layer within the same residuals block, in turn to can achieve a deeper network model. Within the residual network, each building block performs an identity mapping, and the resulting features are element-wise summed across the convolutional layers preceding and following the identity connection. Figure 2 illustrates the foundational architecture of ResNet101. The feature extraction layer of the ResNet101 model produces an output with dimensions of (2048, 7, 7). Subsequently, a 1 × 1 convolutional layer with 1,024 convolutional kernels is added to the base model, which modifies the output dimension to (1,024, 7, 7).

#### 3.3.2 DenseNet121

**Figure 3:**
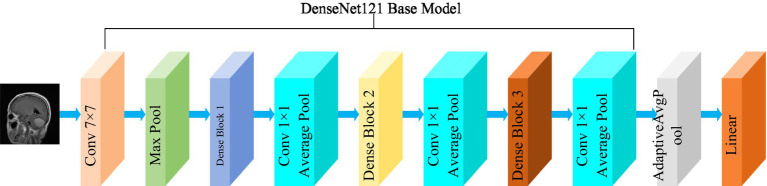
Structure of the DenseNet121 model.

The DenseNet convolutional neural network model was proposed by Huang et al. (2017). The network is based on the ResNet structure, but it incorporates dense connections (i.e., summed variable joins) between all preceding and subsequent layers. Another significant aspect of DenseNet is the reuse of features through channel connections. In DenseNet, every layer receives feature maps as input from all preceding layers, and its output feature maps are subsequently utilized as input for each subsequent layer. In ResNet, the features of each block are combined by summation, whereas in DenseNet, feature aggregation is accomplished through concatenation. Figure 3 shows the fundamental framework of the DenseNet121 model. The core of the network is the reused combination of Dense Blocks and Transition Layers, forming the intermediate structure of DenseNet. Additionally, the topmost part of DenseNet consists of a 7 × 7 convolutional layer with a stride of 2, and a 3 × 3 MaxPool2d layer with a stride of 2. The output dimension of the feature extraction layer of the model is (1,024, 7, 7).

#### 3.3.3 EfficientNetB0

The EfficientNet model was proposed by the Google AI research team in 2019 (Tan and Le, 2019). In contrast to traditional scaling methods used in previous studies, where the width, depth, and resolution of the deep CNN architecture are arbitrarily increased to enhance model performance, EfficientNets achieve network performance improvement through a fixed-scale approach that scales the width, depth, and resolution of the network’s input images. The calculations are as follows [Equations (2–6)]:


(2) $$\mathrm{Depth}:d=\alpha^{\varphi }$$

(3) $$\mathrm{Width}:w=\beta^{\varphi }$$

(4) $$\mathrm{resolution}\;\mathrm{ratio}:r=\gamma^{\varphi }$$

(5) $$s.t.\;\alpha \cdot \beta^{2}\cdot \gamma^{2}\approx 2$$

**Figure 4:**
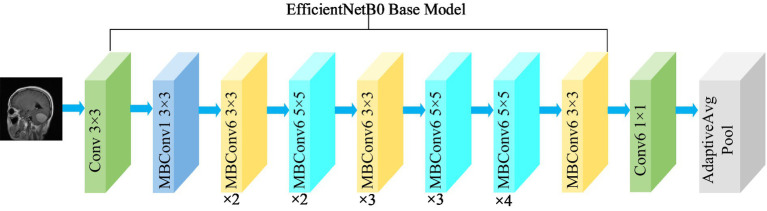
Structure of the EfficientNetB0 model.

(6) α≥1,β≥,γ≥1

where, *α*, *β*, and *γ* are obtained by hyperparametric mesh search techniques and can determine the allocation of additional resources to the width, depth, and resolution of the network. *φ* is a user-specified coefficient that controls the amount of additional resources used for model scaling. In Figure 4, the structure of the EfficientNetB0 model is shown. In order to transform the feature output of the EfficientNetB0 model from its original dimension of (1,280, 7, 7) to the desired dimension of (1,024, 7, 7), a 1×1 convolution with 1,024 convolution kernels is applied so that the output is (1,024, 7, 7).

### 3.4 Training of CNNs

The convolutional neural network training process is a combination of forward and backward propagation. It starts at the input layer and propagates forward from layer to layer until it reaches the classification layer. The error is then propagated back to the first layer of the network. In layer L of the network, input from layer L-1 neuron j is received in a forward propagation path. The weighted sums are calculated as follows [Equation (7)]:


(7) $$\mathrm{In}=\overset{n}{\underset{j=1}{\sum }}W_{ij}^{l}x_{j}+b_{i}$$

Here, the letters *W l ij* stand for weights, *x_j_* stand for training samples, and *b_i_* stand for bias. The nonlinearity of the model can be increased by the activation function to make the network fit the data better. Equation (8) shows how the Relu function is calculated.


(8) $$R_{i}^{l}=\max\left(0,\;In_{i}^{l}\right)$$

In the classification layer of the convolutional neural network, the probability of categorization is calculated by the following softmax function. This classification layer evaluates the probability score of each category by softmax function. Equation (9) shows the method of calculation.


(9) $$out_{i}^{l}=\frac{e^{In_{i}^{l}}}{\sum_{i}e^{out_{i}^{l}}}$$

CNN weights are updated by Backpropagation. The algorithm uses unknown weight *W* to minimize the tracking cost function. The loss function is calculated as follows [Equation (10)]:


(10) $$C=-\frac{1}{m}\overset{m}{\underset{i}{\sum }}\ln\left(P\left(\frac{y_{i}}{x_{i}}\right)\right)$$

Here, *m* represents the total count of training samples. *x_i_* represents the initial training sample. *y_i_* represents the label associated with the sample *x_i_*. And $P\left(\frac{y_{i}}{x_{i}}\right)$ represents the probability of *x_i_* belonging to class *y_i_*.

Stochastic gradient descent on small batches of size *N* is used to minimize the cost function *C* and approximate the training cost by the small batch cost. *W* denotes the weights at iteration t of the l convolutional layer, and *C* denotes the small batch cost. The weights are then updated in the next iteration as follows [Equation (11)]:


$$\gamma^{t}=\gamma^{\left[\frac{tN}{m}\right]}$$

(11) $$V^{t+1}=\mu V_{l}^{t}-\gamma^{t}\alpha_{l}\frac{\partial C}{\partial W}$$

$$W_{i}^{t+1}=W_{l}^{t}+V_{t}^{t+1}$$

In this case, *α_l_* is the learning rate of layer l. *γ* is the scheduling rate that reduces the initial learning rate at the end of a specified number of periods. And *μ* stands for the momentum factor, which indicates the effect of the previously updated weights on the current iteration.

## 4 Results and discussion

The experiments were conducted on a Windows 10 system with 64 GB of Random Access Memory (RAM). The graphics card utilized was RTX 4070, and the programming language employed was Python, with PyTorch serving as the framework. The hyperparameters of the model in the experiment are shown in Table 1.

**Table 1:** Hyperparameters.

Parameters	Setting
Epoch	25
Learning rate	0.0001
Batch size	32
Optimizer	Adam
Loss function	Cross entropy

### 4.1 Evaluation metrics

To comprehensively assess the effectiveness of the model, the evaluation metrics including accuracy, precision, recall, and F1-score are employed in this paper. The expressions of the evaluation metrics are shown in Equations (12–15) (Yeung et al., 2022; Alyami et al., 2023).


(12) $$\mathrm{Accuracy}=\frac{TP+TN}{TP+TN+FP+FN}$$

(13) $$\mathrm{Precision}=\frac{TP}{TP+FP}$$

(14) $$\mathrm{Recall}=\frac{TP}{TP+FN}$$

(15) $$F1-\mathrm{score}=\frac{2\times \mathrm{precision}\times \mathrm{recall}}{\mathrm{precision}+\mathrm{recall}}$$

where, true positive (*TP*) represents the count of accurately classified sick images in each respective category. True negative (*TN*) denotes the total number of correctly classified images in all categories, excluding the relevant category. False negative (*FN*) represents the count of incorrectly classified images in the relevant category. False positive (*FP*) denotes the count of misclassified images in all categories, excluding the relevant category.

### 4.2 Classification results

This section presents the classification results of the proposed method and includes a comparative analysis with and without the utilization of feature fusion methods.

#### 4.2.1 The representation of a single model

**Figure 5:**
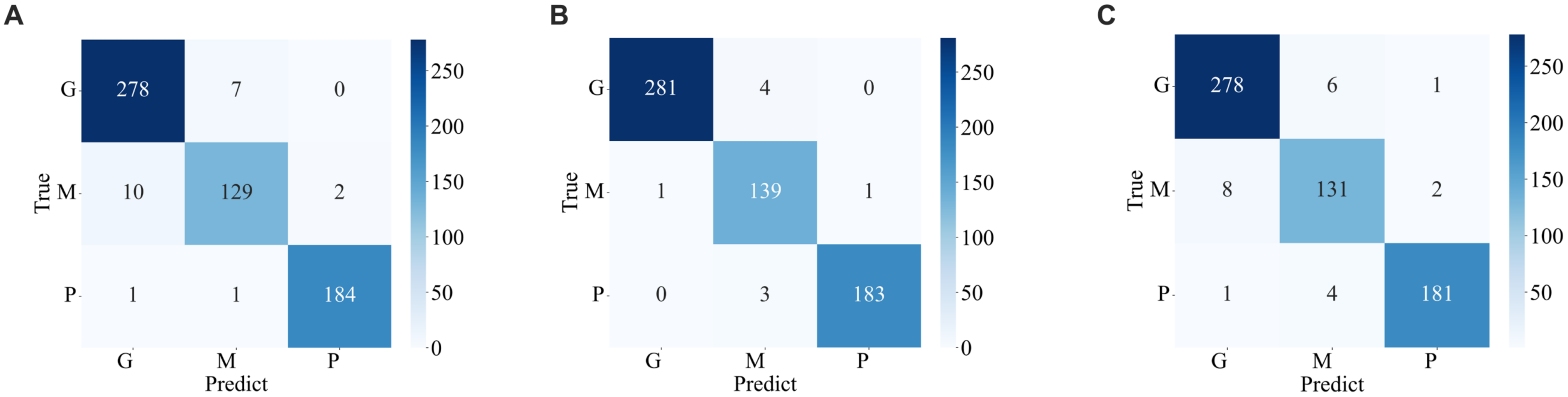
Confusion matrix of predicted results for a single model on the test set of the dataset 1. **(A)** ResNet101 **(B)** DenseNet121 **(C)** EfficientNetB0.

**Figure 6:**
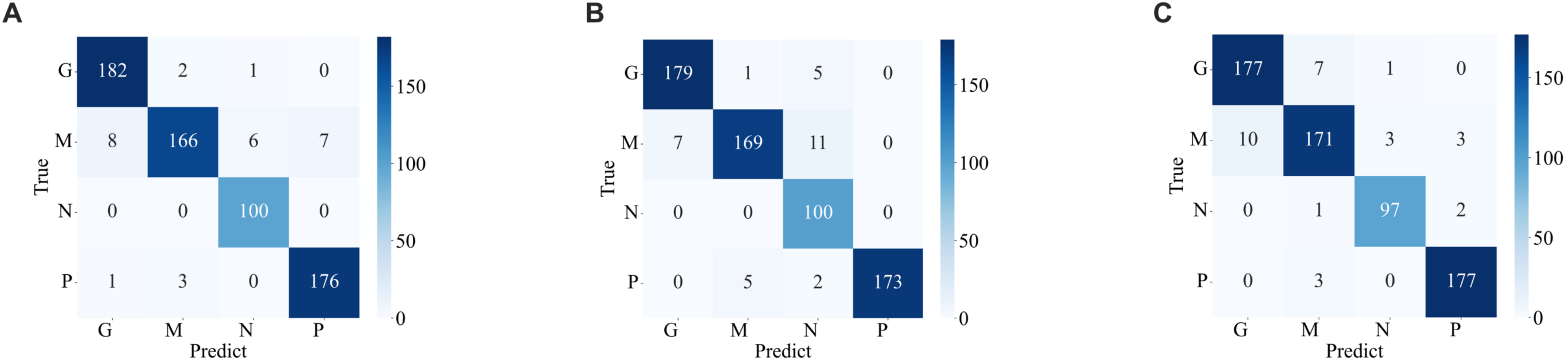
Confusion matrix of the predicted results of a single model on the test set of the dataset 2 **(A)** ResNet101 **(B)** DenseNet121 **(C)** EfficientNetB0.

The confusion matrix illustrating the classification results of models, which was pre-trained through fine-tuning on the test set of the dataset 1, is presented in Figure 5. To analyze the classification outcomes of the three pre-trained models on the test set of the dataset 2, Figure 6 shows the corresponding confusion matrix. Additionally, Table 2 lists the specific values of accuracy, precision, recall, and F1-score, calculated using Equations (12–15) respectively. According to Table 2, on dataset 1, DenseNet121 has the best classification performance for brain tumor with 98.53% accuracy, while on dataset 2, ResNet101 has excellent classification performance with 95.71% accuracy.

**Table 2:** Indicators for the classification of a single model.

Dataset	Model	Tumor type	Precision	Recall	F1-score	Accuracy
Dataset 1	ResNet101	Glioma	96.19%	97.54%	96.86%	
		Meningioma	94.16%	91.49%	92.81%	
		Pituitary	98.92%	98.92%	98.92%	
		average	96.43%	95.99%	96.20%	96.57%
	DenseNet121	Glioma	99.65%	98.60%	99.12%	
		Meningioma	95.21%	98.58%	96.86%	
		Pituitary	99.46%	98.39%	98.92%	
		average	98.10%	98.52%	98.30%	98.53%
	EfficientNetB0	Glioma	96.86%	97.54%	97.20%	
		Meningioma	92.91%	92.91%	92.91%	
		Pituitary	98.37%	97.31%	97.84%	
		average	96.05%	95.92%	95.98%	96.41%
Dataset 2	ResNet101	Glioma	95.29%	98.38%	96.81%	
		Meningioma	97.08%	88.77%	92.74%	
		NoTumor	93.46%	100.0%	96.62%	
		Pituitary	96.17%	97.78%	96.97%	
		Average	95.50%	96.23%	95.78%	95.71%
	DenseNet121	Glioma	96.24%	96.76%	96.50%	
		Meningioma	96.57%	90.37%	93.37%	
		NoTumor	84.75%	100.0%	91.74%	
		Pituitary	100.0%	96.11%	98.02%	
		Average	94.39%	95.81%	94.91%	95.25%
	EfficientNetB0	Glioma	94.65%	95.68%	95.16%	
		Meningioma	93.96%	91.44%	92.68%	
		NoTumor	96.04%	97.00%	96.52%	
		Pituitary	97.25%	98.33%	97.79%	
		Average	95.48%	95.61%	95.54%	95.40%

#### 4.2.2 With feature fusion

**Figure 7:**
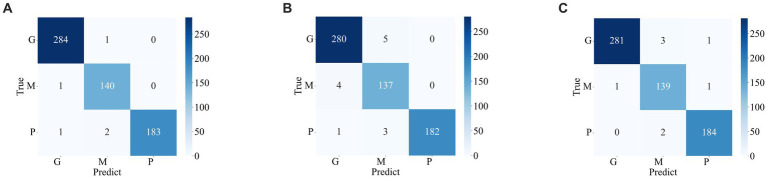
Classification results of brain tumors on the test set of the dataset 1 **(A)** ResNet101 + DenseNet121 **(B)** ResNet101 + efficientNetB0 **(C)** DenseNet121 + EfficientNetB0.

**Figure 8:**
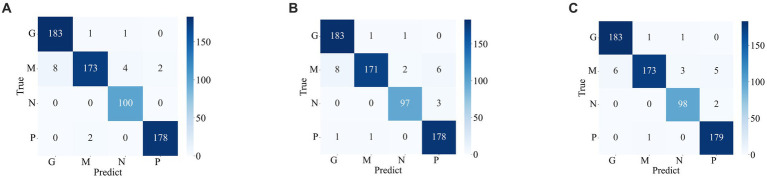
Classification results of brain tumors on the test set of the dataset 2 **(A)** ResNet101 + DenseNet121 **(B)** ResNet101 + efficientNetB0 **(C)** DenseNet121 + EfficientNetB0.

Figures 7, 8 display the confusion matrices of the brain tumor classification results achieved by feature fusion on dataset 1 and dataset 2, respectively. Furthermore, Table 3 present detailed values of the classification indexes for dataset 1 and dataset 2. It can be seen that ResNet101 + DenseNet121 attains optimal classification results on both datasets, with an accuracy of 99.18% on dataset 1 and 97.24% on dataset 2.

**Table 3:** The classification results of feature fusion methods.

Dataset	Model	Tumor type	Precision	Recall	F1-score	Accuracy
Dataset 1	ResNet101 + DenseNet121	Glioma	99.30%	99.65%	99.47%	
		Meningioma	97.90%	99.29%	98.58%	
		Pituitary	1.00%	98.39%	99.19%	
		average	99.07%	99.11%	99.08%	99.18%
	ResNet101 + EfficientNetB0	Glioma	98.25%	98.25%	98.25%	
		Meningioma	94.48%	97.16%	95.80%	
		Pituitary	1.00%	97.85%	98.91%	
		average	97.58%	97.75%	97.65%	97.88%
	DenseNet121 + EfficientNetB0	Glioma	99.65%	98.60%	99.12%	
		Meningioma	96.53%	98.58%	97.54%	
		Pituitary	98.92%	98.92%	98.92%	
		average	98.37%	98.70%	98.53%	98.69%
Dataset 2	ResNet101 + DenseNet121	Glioma	95.81%	98.92%	97.34%	
		Meningioma	98.30%	92.51%	95.32%	
		NoTumor	95.24%	1.00%	97.56%	
		Pituitary	98.89%	98.89%	98.89%	
		Average	97.06%	97.58%	97.28%	97.24%
	ResNet101 + EfficientNetB0	Glioma	95.31%	98.92%	97.08%	
		Meningioma	98.84%	91.44%	95.00%	
		NoTumor	97.00%	97.00%	97.00%	
		Pituitary	95.19%	98.89%	97.00%	
		Average	96.59%	96.56%	96.52%	96.47%
	DenseNet121 + EfficientNetB0	Glioma	96.83%	98.92%	97.86%	
		Meningioma	98.86%	92.51%	95.58%	
		NoTumor	96.08%	98.00%	97.03%	
		Pituitary	96.24%	99.44%	97.81%	
		Average	97.00%	97.22%	97.07%	97.09%

**Figure 9:**
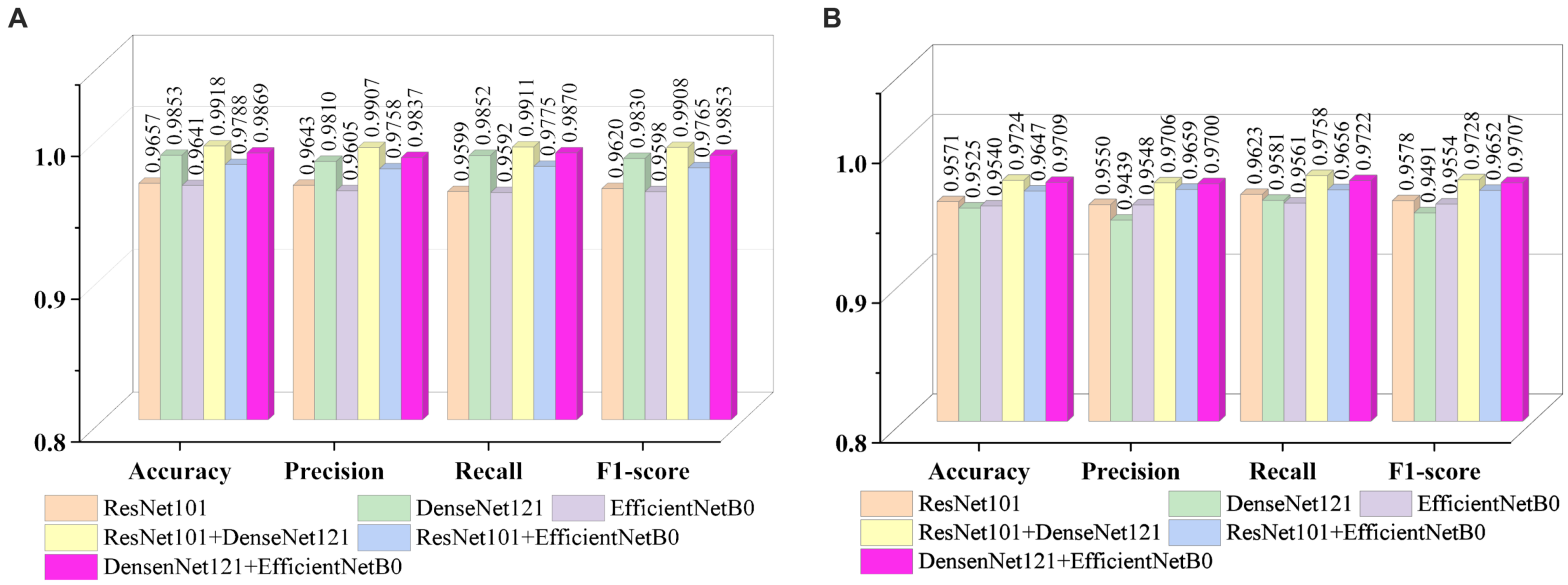
Visualization of brain tumor classification metrics **(A)** dataset 1 **(B)** dataset 2.

Figures 9A, B show the average evaluation metrics for brain tumor classification of every model on dataset 1 and dataset 2, respectively. On the dataset 1, from Figure 9A, it can be observed that the combination of ResNet101 and DenseNet121 (ResNet101 + DenseNet121) achieved the best classification accuracy, precision, recall, and F1-score, with values of 99.18, 99.07, 99.11, and 99.08%, respectively. Additionally, among the individual models, EfficientNetB0 exhibits the best classification results for brain tumor classification. Notably, DenseNet121 outperforms ResNet101 + EfficientNetB0 but is outperformed by both ResNet101 + DenseNet121 and DenseNet121 + EfficientNetB0. In Figure 9B (i.e., dataset 2), the ResNet101 + DenseNet121 model also achieves the best performance. However, among the individual models, DenseNet121 exhibits the best classification results, with accuracy, precision, recall, and F1-score of 97.24, 97.06, 97.58, and 97.28%, respectively. Unlike dataset 1, where DenseNet121 showed strong performance, it appears to have the weakest classification ability on the dataset 2. Conversely, ResNet101 + DenseNet121, ResNet101 + EfficientNetB0, and DenseNet121 + EfficientNetB0 all outperform the individual models. The experimental results validate the effectiveness of combining features from different models through feature fusion, thus providing a more reliable approach for brain tumor classification than relying on a single model. In addition, the average improvement of ResNet101 + DenseNet121 is 2.085% (dataset 1 is 2.61%, dataset 2 is 1.56%) and 1.32% (dataset 1 is 0.65%, dataset 2 is 1.99%) compared with ResNet101 and DenseNet121, respectively. Similarly, the accuracy improvement for ResNet101 + EfficientNetB0 is 1.035% (1.31% for dataset 1 and 0.76% for dataset 2) and 1.345% (1.47% for dataset 1 and 1.22% for dataset 2) compared with ResNet101and EfficientNetB0 alone. In comparison with Densenet121 and EfficientNetB0, the average accuracy improvement for DenseNet121 + EfficientNetB0 is 1.225% (0.61% for dataset 1 and 1.84% for data set 2) and 1.985% (2.28% for dataset 1 and 1.69% for dataset 2), respectively. The modeled results strongly support the efficacy of employing feature fusion in brain tumor classification. In addition, it is evident that ResNet101 achieves the most favorable classification results, while DenseNet121 yields the terrible results on dataset 2. But the classification effectiveness of ResNet101 + DenseNet121 surpasses that of ResNet101 + EfficientNetB0 and DenseNet121 + EfficientNetB0. This suggests that the combination of ResNet101 and DenseNet121 outperforms configurations involving EfficientNetB0. The possible reason for this phenomenon is the inferior feature matching effect of ResNet101 + EfficientNetB0 and DenseNet121 + EfficientNetB0 compared to ResNet101 + DenseNet121.

**Figure 10:**
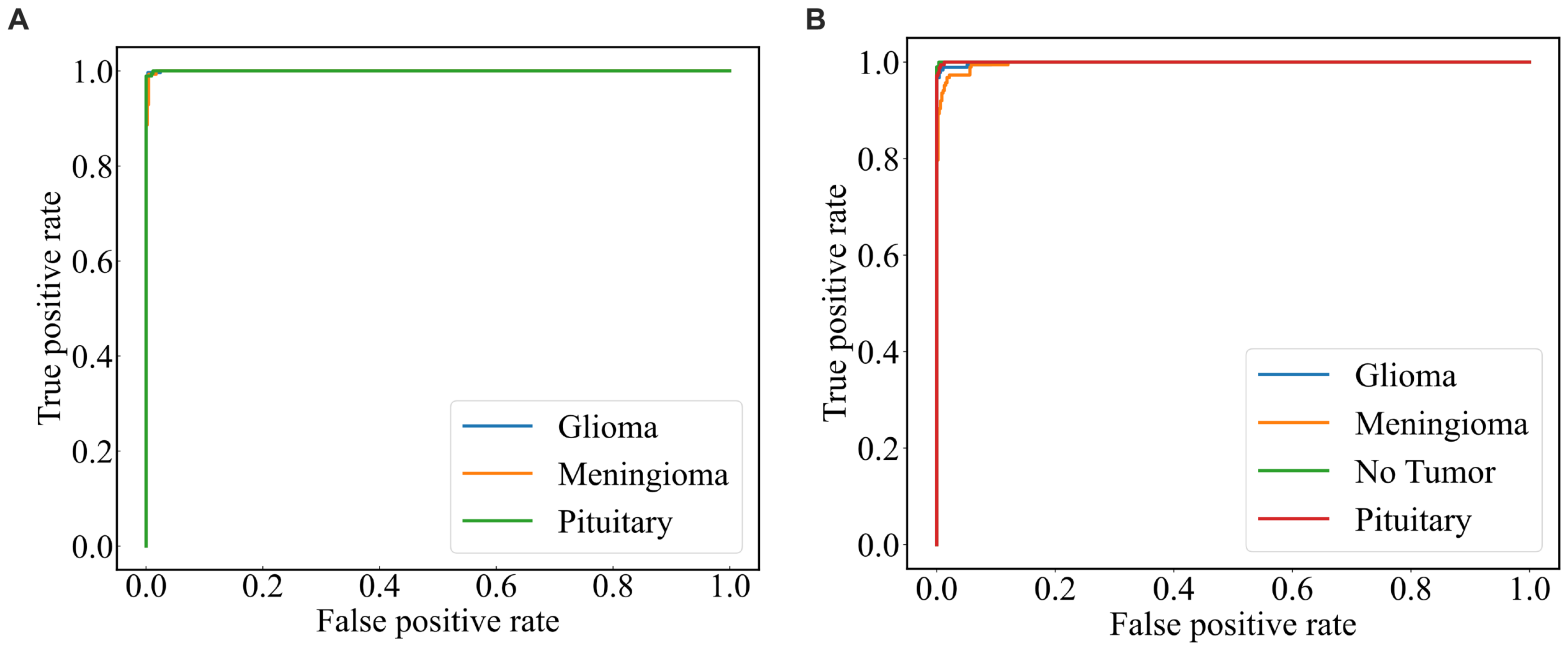
ROC curve of the model **(A)** dataset 1 **(B)** dataset 2.

A subject Receiver Operating Curve (ROC) is also utilized in the analysis process. It is a curve that illustrates the relationship between the true positive rate and the false positive rate. The size of the Area Under Curve (AUC) of the ROC curve indicates the strength of the model’s ability to differentiate between different types of tumors, with a larger AUC value indicating better classification performance. As shown in Figure 10, the ROC curves of ResNet101 + DenseNet121 for the model are demonstrated and the values of AUC for the three types of brain tumors in dataset 1 are 0.9987, 0.9952, and 0.9999, respectively. In dataset 2, the values of AUC are 0.9991, 0.9971, 0.9999, and 0.9998, respectively.

#### 4.2.3 Cross-dataset validation and robustness validation

Based on the foregoing, it is evident that the ResNet101 + DenseNet121 yields superior classification results across the two public datasets. This section aims to assess the robustness of ResNet101 + DenseNet121. To further assess the model’s robustness, a cross-data verification method was employed. The normal class in Dataset 2 was excluded, and data from the remaining three brain tumor classes were utilized to evaluate the dataset 1 trained model, ResNet101 + DenseNet121. The precision, recall, F1-score and accuracy of ResNet101 + DenseNet121 are verified to be 94.71, 94.44, 94.41, and 94.38%, respectively, which indicates its good robustness.

### 4.3 Discussion

There have been many studies on brain tumor classification. Among these methods, the key is the extracted features. Generally, there is a relationship between the effectiveness of the model and the amount of data. Whereas the acquisition of medical images is usually difficult and expensive. Transfer learning can take full advantage of its advantages on tasks with small datasets to improve model performance, accelerate the training process, and reduce the risk of overfitting. In addition, model integration is a technique that combines the prediction results of multiple independently trained models to obtain more powerful and robust global predictions, which can improve the upper limit of performance. In our work, the pre-trained model is used to extract the features of the image, and then the extracted features are fused using the model integration method of feature fusion to enhance the ability of the model.

**Figure 11:**
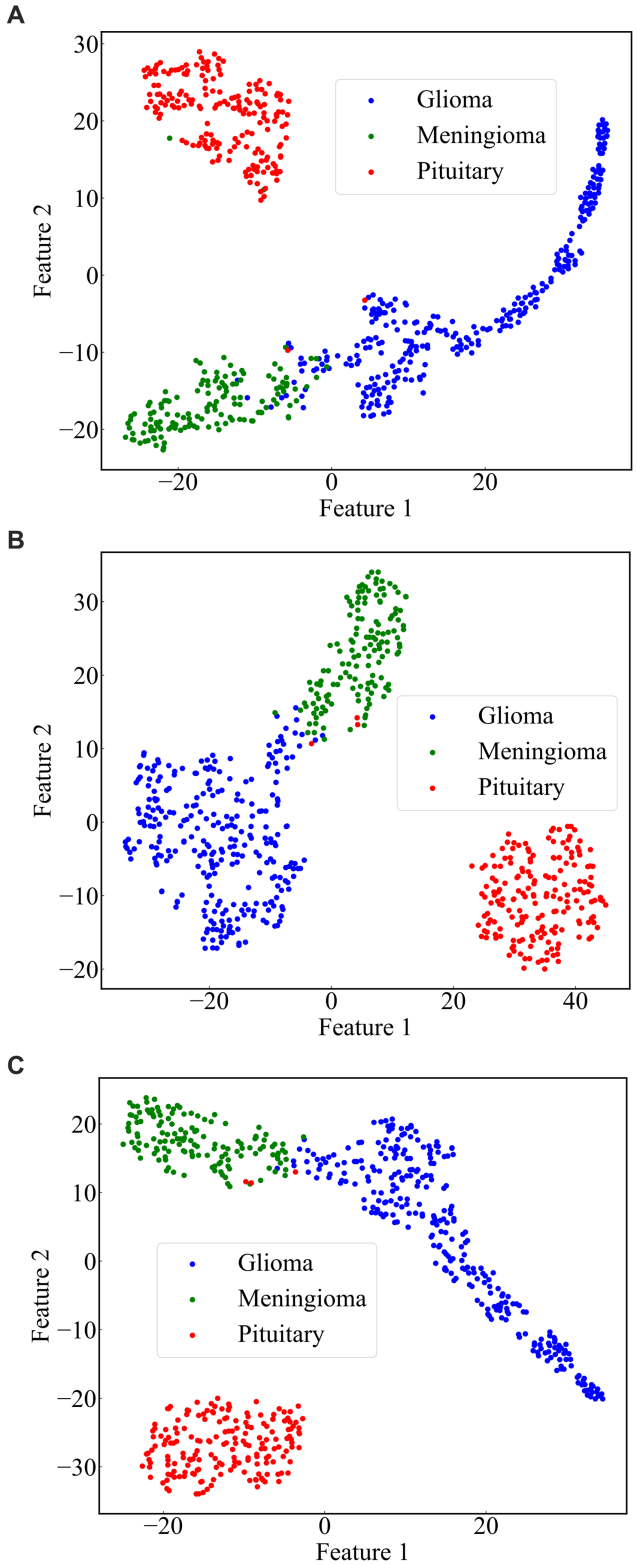
Scatterplot of the feature set. **(A)** ResNet101 **(B)** DenseNet121 **(C)** ResNet101 + DenseNet121.

From the previous analysis, it can be found that among the three fused models, ResNet101 + DenseNet121 achieves the best classification results. ResNet101 adopts the method of residual learning to construct residual blocks, which makes the network easier to train and reduces the problem of gradient vanishing. Densenet121, on the other hand, uses the idea of dense connectivity, where each layer’s input contains the output of all previous layers. This kind of connection is helpful to the transmission of information and the flow of gradients, and slows down the problem of information bottleneck. Dense connectivity also facilitates feature reuse. The features extracted by ResNet101 and those extracted by Densenet121 are fused to realize the complementary feature, which makes the feature more abundant and diversified, and thus achieves better classification effect. To demonstrate the effectiveness of the proposed method, we use the method of t-Distributed Stochastic Neighbor Embedding (t-SNE) to visualize the features extracted by the model ResNet101 + DenseNet121 trained on dataset 1, and the visualization results are shown in Figure 11. The feature set of ResNet101 is shown in Figure 11A. It can be seen that some gliomas and meningiomas are nested with each other. The mean and standard deviation of the feature set are−0.0057 and 0.6141, respectively. The feature set of DenseNet121 is shown in Figure 11B, which shows that only a few gliomas and meningiomas are nested with each other. The mean and standard deviation of the feature set are 0.2323 and 0.652795, respectively. Figure 11C displays the feature set of ResNet101 + DenseNet121, indicating minimal nested classes. The mean and standard deviation of the feature set are 0.2267 and 0.9604, respectively. Additionally, the analysis shows that the standard deviation of the feature set of ResNet101 + Densenet121 is the highest, which also shows that ResNet101 + Densenet121 increases the uniqueness of extracting the image features of brain tumors and enhances the ability to distinguish brain tumors.

### 4.4 Comparison with other state of the art methods

We compared the classification results obtained in this study with those reported in the literature using the same dataset. The compared results shown in Table 4 demonstrate that our study achieved competitive classification performance when compared to the state-of-the-art approaches in the current literature.

**Table 4:** Comparison with other state-of-the-art models.

Reference	Dataset	Method	Accuracy
Gumaei et al. (2019)	Dataset 1	RELM	94.233%
Swati et al. (2019)	Dataset 1	Fine-tuning the VGG19 model.	94.82%
Ghassemi et al. (2020)	Dataset 1	Pre-trained GAN	95.6%
Deepak and Ameer (2021)	Dataset 1	CNN + SVM	98%
Sekhar et al. (2022)	Dataset 1	GoogLeNet+KNN	98.3%
Öksüz et al. (2022)	Dataset 1	Deep and shallow feature fusion	97.25%
Hammad et al. (2023)	Dataset 1	CNN model with 8 layers	96.86%
Saurav et al. (2023)	Dataset 1	Pre-trained ResNet50	97.48%
Kang et al. (2021)	Dataset 2	Feature connection	91.8%
Demir and Akbulut (2022)	Dataset 2	R-CNN	96.6%
Hossain et al. (2023)	Dataset 2	Ensemble Model	96.94%
Alanazi et al. (2022)	Dataset 1	CNN	96.89%
	Dataset 2		95.75%
Saurav et al. (2023)	Dataset 1	AG-CNN	97.23%
	Dataset 2		95.71%
Proposed model	Dataset 1	ResNet101 + DenseNet121	99.18%
	Dataset 2		97.24%

## 5 Conclusion

This paper proposes a novel method for brain tumor classification, utilizing feature fusion to improve performance. Three advanced pre-trained models including ResNet101, DenseNet121, and EfficientNetB0, were selected as base models and adjusted to have the same output size (1,024, 7, 7). Brain tumor images were fed into these models to extract their respective features, and then feature fusion was achieved by pairwise combination of the models through feature summation. The fused features were subsequently used for the final classification. The method was validated on two publicly available datasets, and evaluation metrics such as accuracy, precision, recall, and F1-score were employed. Experimental Results indicated that the combination of ResNet101 and DenseNet121 (ResNet101 + DenseNet121) achieved the best classification results for both dataset 1 and dataset 2. On dataset 1, accuracy of 99.18%, precision of 99.07%, recall of 99.11%, and F1-score of 99.08% were achieved. For dataset 2, the corresponding metrics values including accuracy of 97.24%, precision of 97.06%, recall of 97.58%, and F1-score of 97.28% were obtained. Comparing our method with other state-of-the-art techniques, our approach exhibits superior classification performance. In the future, we plan to study two important works. On one hand, we will expand the experimentation by incorporating additional models to validate the effectiveness of feature fusion through summation for brain tumor classification. On the other hand, we aim to extend this method to encompass other brain diseases, thus enhancing the model’s capacity to recognize multiple classes of brain diseases.

## Data availability statement

Publicly available datasets were analyzed in this study. This data can be found here: https://figshare.com/articles/dataset/brain_tumor_dataset/1512427 and https://www.kaggle.com/datasets/sartajbhuvaji/brain-tumor-classification-mri.

## Author contributions

WC: Formal analysis, Software, Validation, Visualization, Writing – review & editing. XT: Software, Writing – original draft. JZ: Conceptualization, Investigation, Methodology, Project administration, Writing – original draft. GD: Investigation, Project administration, Visualization, Writing – review & editing. QF: Validation, Writing – review & editing. HJ: Investigation, Methodology, Writing – review & editing.
